# The Potential Utility of Tirzepatide for the Management of Polycystic Ovary Syndrome

**DOI:** 10.3390/jcm12144575

**Published:** 2023-07-10

**Authors:** Alekya Devi Anala, Insiya Sajjad Hussain Saifudeen, Maryam Ibrahim, Moksha Nanda, Nida Naaz, Stephen L. Atkin

**Affiliations:** School of Medicine, Royal College of Surgeons in Ireland Bahrain, Adliya 15503, Bahrain; 20203265@rcsi-mub.com (A.D.A.); 21200444@rcsi-mub.com (I.S.H.S.); 21200503@rcsi-mub.com (M.I.); 21202073@rcsi-mub.com (M.N.); 20200884@rcsi-mub.com (N.N.)

**Keywords:** polycystic ovary syndrome, insulin resistance, obesity, metabolic dysfunction, tirzepatide, glucagon-like peptide receptor agonist, weight loss, hyperandrogenism

## Abstract

Polycystic ovary syndrome (PCOS) is the most prevalent endocrinopathy in women of reproductive age. The metabolic dysfunction associated with PCOS increases the probability of developing type 2 diabetes (T2D), endometrial cancer, and cardiovascular disease. Research has shown that the metabolic features of PCOS may be improved by weight loss following treatment with glucagon-like peptide-1 receptor (GLP-1R) agonists. Tirzepatide is a dual GLP-GIP (gastric inhibitory polypeptide) receptor agonist that shares a very similar mechanism of action with GLP-1R agonists, and it is hypothesized that it may be a potential contender in the treatment of PCOS. The success of GLP-1R agonists is usually hindered by their adverse gastrointestinal effects, leading to reduced compliance. The mechanism of action of Tirzepatide partly addresses this issue, as its dual receptor affinity may reduce the intensity of gastrointestinal symptoms. Tirzepatide has been licensed for the treatment of type 2 diabetes and given the metabolic issues and obesity that accompanies PCOS, it may be of value in its management for those PCOS patients who are obese with metabolic syndrome, although it may not benefit those who are of normal weight. This study reviews the current therapies for the treatment of PCOS and evaluates the potential use of Tirzepatide to address the symptoms of PCOS, including reproductive dysfunction, obesity, and insulin resistance.

## 1. Introduction

Polycystic ovary syndrome (PCOS) is linked with infertility, hypertension, diabetes, an increase in cardiovascular disease, and reduced quality of life [1]. Affecting 5–10% of women, PCOS is the most prevalent endocrine abnormality in premenopausal women [2]. PCOS is a diagnosis of exclusion that can be determined by the National Institute of Health, the Androgen Society, or the Rotterdam criteria [3]. Due to the differing clinical presentations of these women and the complex pathophysiology of this syndrome [4], it is estimated that 70% of women with PCOS in the community are undiagnosed [5].

Anovulation, oligo-ovulation, and hyperandrogenism are defining characteristics of PCOS and are fundamental components common to the different diagnostic criteria [6]. Additionally, a number of distinct clinical manifestations may be apparent, including hirsutism and amenorrhea, together with metabolic disorders such as insulin resistance, prediabetes, type 2 diabetes, and risk of cardiovascular disease [7].

Although the etiology of PCOS remains unknown, it is hypothesized to have emerged due to the interaction between environmental and genetic factors. Consequently, the goal of finding an effective treatment for this metabolic dysfunction has become a challenge. Tirzepatide, a GLP/GIP receptor agonist, has therapeutic potential to address the hallmark features of PCOS in those that are obese, making it a potentially strong candidate for metabolic management.

## 2. Common PCOS Features

**Figure 1:**
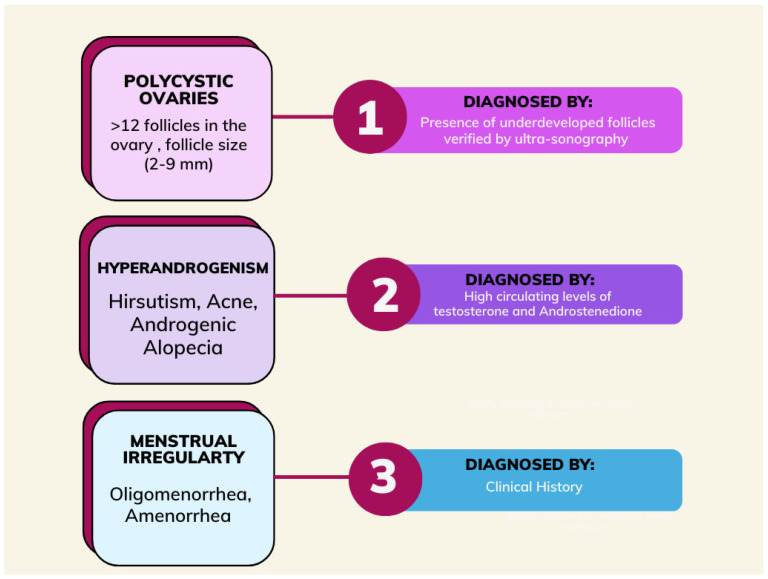
Schematic of the diagnosis of polycystic ovary syndrome.

In accordance with the Rotterdam criteria, polycystic ovary syndrome is defined based on the presence of two of the three criteria after other pathologies, such as congenital adrenal hyperplasia, thyroid abnormalities, and hyperprolactinemia, have been excluded. The Rotterdam criteria include irregular menses, hyperandrogenism, and ovarian volume greater than or equal to 10 mL with or without 12 or more peripheral antral follicles ranging from 2–9 mm in one or both ovaries [8]. In women with PCOS, symptoms do not appear until puberty (Figure 1) [9].

### 2.1. Hyperandrogenism

In PCOS, ovarian androgen production is responsible for clinical and biochemical hyperandrogenemia [10]. Androgens are sex hormones responsible for the development of secondary sexual male characteristics and steroid hormone production, which is important in both males and females [11]. Hyperandrogenism is characterized by elevated total or free testosterone levels, increased androstenedione secretion from the ovaries, or increased dehydroepiandrosterone sulfate secretion from the adrenal gland (DHEAS, an androgen marker for adrenal function). Higher levels of DHEAS were seen in 20–30% of women with PCOS, indicating that hyperandrogenemia is a consequence of hypersecretion from both the ovaries and the adrenal glands [12].

**Figure 2:**
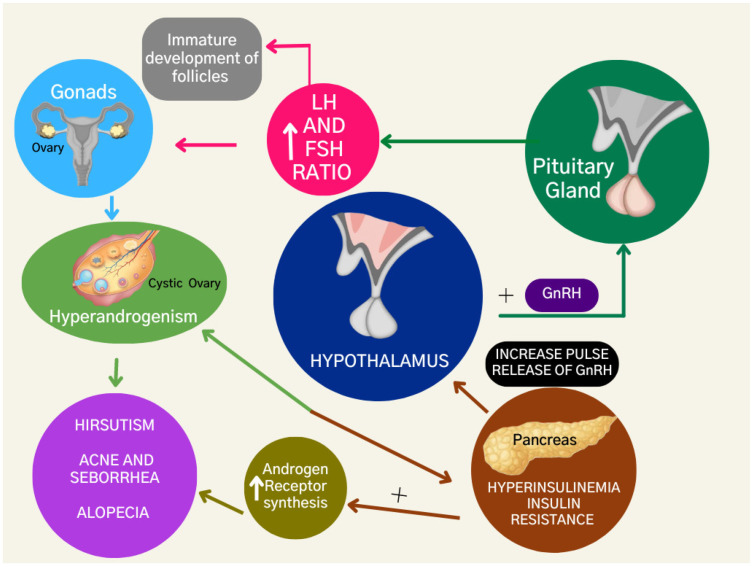
Hormonal and metabolic features of polycystic ovary syndrome.

The dysregulation of steroid hormone production enhances androgen levels due to the dysfunction of granulosa aromatase activity, a crucial enzyme in converting androgens to estrogens [13]. Additionally, the excess luteinizing hormone (L.H.) stimulates the production of androgens in the ovary, while the relative deficiency of follicle-stimulating hormone (F.S.H.) impairs the growth of follicles (Figure 2). As a result, there is an imbalance of the LH:FSH ratio and ovarian theca cells are stimulated to proliferate, resulting in increased steroidogenesis and eventually leading to hyperandrogenism in women with PCOS [14].

Hyperandrogenism presents with the clinical symptoms of acne, hirsutism, and androgenic alopecia. High levels of androgens can also contribute to weight gain, menstrual irregularity, and acanthosis nigricans [15]. A high body mass index, hyperandrogenism, infertility, and hirsutism have all been reported as major contributing factors to depressive symptoms and anxiety in women with PCOS. As a result, patients may withdraw from social interaction and experience mood disturbances [16].

### 2.2. Insulin Resistance

Insulin resistance affects 30–40% of PCOS patients and contributes to the pathophysiology of reproductive dysfunction [17]. Insulin-resistant women with PCOS produce insulin and regulate their blood glucose levels; however, the body fails to respond to insulin efficiently [18] and this may ultimately result in impaired glucose tolerance and type 2 diabetes. This was shown in a study of 254 PCOS patients, of whom 31% had impaired glucose tolerance and 7.5% had type 2 diabetes, compared to non-obese PCOS women of whom 10.3% had impaired glucose tolerance and 1.5% had diabetes. Up to 40% of women with PCOS develop type 2 diabetes or impaired glucose tolerance, and they usually have a family history of diabetes [19].

### 2.3. Hyperinsulinemia

Insulin resistance leads to compensatory hyperinsulinemia [20]. Hyperinsulinemia is diagnosed when plasma insulin levels are greater than 2 µU/mL and the serum glucose concentration is less than 60 mg/dL [21]. Hyperinsulinemia is also associated with ovarian and adrenal hyperandrogenism, as insulin can stimulate androgen production in the ovaries and adrenal glands [22] (Figure 2).

### 2.4. Obesity

The prevalence of obesity is recognized as being at epidemic levels globally [23]. Women with PCOS frequently experience significant weight gain, which is a compounding factor as it also contributes to the pathogenesis of their reproductive issues. Obesity causes and aggravates insulin resistance, as observed in a study that showed a greater prevalence of insulin resistance (64%) reported in obese women than in non-obese (20%) women with PCOS [24]. Moreover, obesity-induced hyperinsulinemia may promote hyperandrogenism in these women [25].

A positive correlation has been observed between weight gain and the number of adipocytes present. Consequently, the increased aromatization of androgens leads to an increased concentration of estrogens within the body. This triggers a negative feedback loop, suppressing gonadotropin production, hence contributing to infertility in women with PCOS. This indicates that obesity contributes to the pathophysiology of PCOS, provoking a vicious cycle [23].

Insulin resistance and hyperandrogenism directly affect each other and can exacerbate hyperandrogenism through actions on the hypothalamus. They can also increase androgen receptor synthesis, which worsens the clinical presentation of hyperandrogenism with hirsutism, acne, seborrhea, and androgenic alopecia.

## 3. PCOS Management

The Endocrine Society guidelines recommend lifestyle changes and weight loss as the initial advice for managing women with PCOS; this includes engaging in physical activity, eating a healthy diet, and making behavioral changes [26]. PCOS treatment generally concentrates on managing infertility due to anovulation and treating symptoms concerning elevated androgen levels, which may require long-term therapy. This approach helps address the issues associated with PCOS (primarily menstrual disorders, acne, obesity, and hirsutism) [27]. Medical pharmacological agents such as liraglutide, metformin, and orlistat have also been used [28].

The treatment paradigm is changing as women globally face different issues regarding PCOS. PCOS treatments may target the GLP-1R as the “anchor pharmacophore”, and there is emerging evidence for using tirzepatide to manage obesity and insulin resistance [29]. Tirzepatide is a dual glucagon-like peptide-1 and glucose-dependent insulinotropic peptide (GIP) receptor agonist and is also known as a “twincretin”. This drug has shown potential through its ability to utilize particular signaling pathways and possesses distinct potencies for receptors involved in obesity and insulin resistance. Therefore, it is hypothesized that tirzepatide should enhance the efficacy that is usually exhibited by GLP-1R agonists [30].

### Lifestyle

Women with PCOS should lose 5–10% of their body weight in order for there to be a notable clinical improvement [31]. Before resorting to pharmacotherapy, lifestyle change/management is recommended to treat obesity in women with PCOS [32]. A healthy diet can lead to weight loss, improved insulin metabolism, increased regularity of menses, and a reduction in testosterone and cholesterol levels [33], but these diets have not been effective in treating biochemical hyperandrogenism [34]. Whilst more research is needed to determine the optimal dietary approach, any diet must focus on delivering a caloric deficit to enable weight loss [31].

In addition to a healthy diet, physical exercise is also recommended for improving clinical outcomes [33]. In a randomized controlled trial involving 130 morbidly obese women with PCOS, exercise reduced waist circumference and liver fat mass more effectively compared to a control group of women with PCOS who did not exercise [35]. Although exercise can lead to improvements in the cardiometabolic profile, it is advised to approach physical activity in a moderate and sustained manner. This is because acute strenuous exercise can activate platelets and lead to negative cardiovascular sequelae. Additionally, practicing intense exercise and extreme dieting may be hazardous [36]. Obese women also suffer from joint problems and arthritis; therefore, exercise therapy may be limited for these subjects [31].

Changes in behavioral habits can also aid women in accomplishing their weight loss goals, which further promotes the alleviation of some PCOS symptoms. When it comes to addressing mental wellbeing, creating goals and having a support group are common approaches. Moreover, the implementation of self-help routines, such as ensuring adequate sleep, can reduce stress in PCOS patients [37]. The frequent occurrence of depressive symptoms (44%) and anxiety (42.9%) in PCOS patients causes them to experience lower moods and loss of motivation [38]. Consequently, this can make these lifestyle changes extremely challenging to implement and maintain [31,38]. This has translated into the low adherence rates reported amongst PCOS patients enrolled in lifestyle intervention programs, which focus on a hypocaloric diet, exercise, and behavioral strategies [39].

## 4. PCOS Pharmacotherapy

### 4.1. Glucagon-Like Peptide-1 Receptor Agonists

Studies have demonstrated that substantial weight loss will only occur with a caloric deficit, where energy expenditure exceeds energy intake [40].

**Figure 3:**
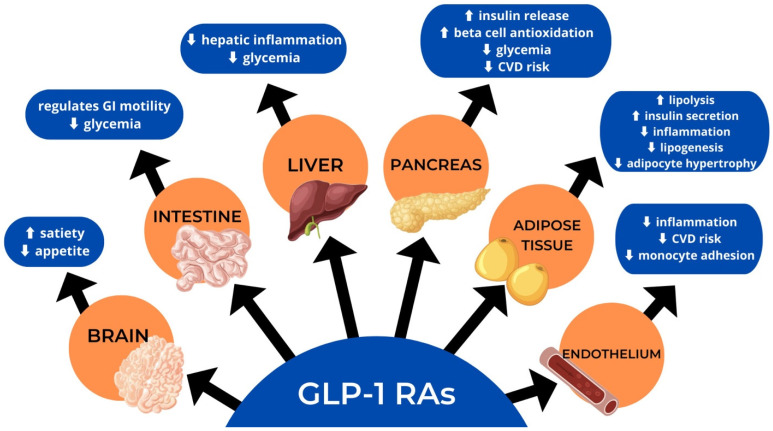
Actions of glucagon-like peptide-1 receptor agonists (GLP-RA).

GLP-1R agonists are tailored to emulate the effects of the hormone GLP-1, secreted from the L cells in the distal portion of the small intestine [41], which causes a reduction in glucagon release, suppression of the hypothalamic hunger center, and slowing of gastric emptying (Figure 3). In addition, GLP-1R agonists affect insulin release, increasing postprandial glucose levels that lead to early satiety [42].

GLP-1R agonists are well recognized for reducing energy intake by suppressing appetite and achieving a state of satiety [43]. GLP-1R immunoreactivity was found in the neurons of the myenteric and submucosal plexuses within the duodenum and proximal colon in animal models. These receptor populations regulate gastrointestinal motility [43]. Further investigation established that neuronal GLP-1R located within the brain mediates the anorectic effect of GLP-1R agonists [44]. The precise location of these receptors in the brain was determined through the use of fluorescent-tagged liraglutide, a GLP-1R agonist, that was tracked after subcutaneous injection in mice. The receptors were observed to be located within structures influencing feeding behavior, like the hypothalamus, brainstem, and circumventricular organs like the median eminence and area postrema [45].

GLP-1R agonists also regulate homeostatic feeding, which involves adjusting caloric intake to maintain energy balance. This is achieved by enhancing the functional connectivity between the nucleus tractus solitarius and the arcuate nucleus within the hypothalamus. These structures are a crucial part of the main regulatory circuit of homeostatic feeding [46].

### 4.2. The Effect of GLP-1R Agonists on Lipid Metabolism

At a molecular level, a correlation is seen between insulin resistance and lipid metabolism disorders. Insulin sensitivity can be negatively affected by an elevation in the levels of cholesterol, triglycerides, low-density lipoproteins, and very low-density lipoproteins [47]. The GLP-1R agonist liraglutide was shown to reduce triglycerides, total cholesterol, and non-esterified fatty acid levels. Reduced concentrations of leptin and elevated levels of adiponectin were also observed [48]. This indicates that GLP-1R agonists play a major role in lipid metabolism [49] and can be optimized when treating dyslipidemia [47]. It is believed that these agonists function by modulating micro-RNA involved in lipid metabolism and inducing lipid metabolic enzymes [50].

Adipogenesis is a process used to describe the hypertrophy of adipocytes and results in the accumulation of adipose tissue [51]. GLP-1R agonists limit the hypertrophy of adipocytes by inducing the phosphorylation of specific transcription factors, such as cAMP response element-binding protein (CREB) and extracellular signal-regulated kinase (ERK). This decreases the expression of fatty acid synthase, an enzyme crucial in lipogenesis [52]. Consequently, a significant reduction in visceral fat may follow [49]. A study on liraglutide showed a reduction in the accumulation of triglycerides in the liver and mesenteric adipose tissue [48]. In addition, liraglutide also caused a reduction of hepatic lipid deposition due to the downregulation of the expression of monoacylglycerol and diglyceride acyltransferases, key enzymes in triglyceride synthesis.

GLP-1R agonists promote lipolysis by stimulating the fibronectin type III domain-containing protein 5 (FNDC5) gene in pancreatic beta cells. Activation of this gene also enhances insulin secretion. Therefore, the balance between glucose and insulin levels leads to lower plasma cholesterol, triglycerides, and free fatty acids [53].

### 4.3. The Effect of GLP-1R Agonists on Oxidative Stress

Oxidative stress is hypothesized to play a major role in the pathophysiology of PCOS [54]. Oxidative stress is commonly described as the imbalance between free radicals and antioxidants within the body [55,56]; it has been closely correlated to the PCOS phenotype of obesity and insulin resistance [54] while also increasing the probability of cardiovascular disease in women with PCOS [57]. It has been reported that women with PCOS had a 19% higher probability of developing cardiovascular disease compared to patients without PCOS [58]. Additionally, lower levels of glutathione and total antioxidant capacity were observed in PCOS women [54].

Liraglutide may reduce oxidative stress, an effect independent of its effect on glucose metabolism. However, importantly, this study was done on patients with type 2 diabetes, not women with PCOS [59]. The GLP-1 hormone enhances the capability of pancreatic beta cells to antioxidize by activating the transcription factor nuclear factor erythroid 2-related factor 2 (NRF2) [60], which expresses genes involved in cellular defense against oxidative stress [61]. Antioxidation with the aid of GLP-1 is also achieved by inactivating ERK 1/2, another transcription factor involved in promoting oxidative stress and intracellular reactive oxygen species [62].

By reducing oxidative stress, GLP-1R agonists prevent damage to endothelial cells from endothelial dysfunction, which impedes atherosclerosis development [62], further decreasing the risk of cardiovascular disease [63].

### 4.4. The Effect of GLP-1R Agonists on Inflammation

Inflammation plays a significant role in the pathogenesis of PCOS [64]. Hence, therapies based around GLP-1 may be more efficient in treating PCOS because of their anti-inflammatory properties [65]. Inflammation is often exacerbated by an increase in visceral adiposity and the pro-inflammatory impact of hyperandrogenism [65].

When monocytes migrate from blood vessels into the interstitial space, inflammation is provoked in adipocytes. These monocytes develop into macrophages once the adipocytes have produced monocyte chemoattractant protein-1 (MCP-1), a pro-inflammatory cytokine. The migration of these monocytes is more vigorous in individuals with obesity; however, animal studies have demonstrated that GLP-1 causes a decline in the infiltration of macrophages within adipose tissue [66] and reduces the production and expression of pro-inflammatory cytokines, such as interleukin-6 (IL-6), tumor necrosis factor-alpha (TNF-α), and MCP-1 [67].

The anti-inflammatory characteristics of this pharmaceutical class were further suggested by a cell line study using exendin-4, a GLP-1R agonist. Exendin-4 inhibited nuclear factor kappa B (NF-κB), a transcription factor associated with the response to inflammation. Consequently, the suppression of lipopolysaccharide (LPS)-induced monocyte migration and pro-inflammatory cytokine secretion was observed. This GLP-1R agonist also caused a rise in insulin-stimulated glucose uptake [68,69] and further exerted its anti-inflammatory effects by increasing the secretion and production of the hormone adiponectin [70], which has both anti-inflammatory and insulin-sensitizing properties [71]. Exenatide therapy in PCOS patients led to weight loss and an improvement in inflammatory markers [72].

Other tissues, like the vascular endothelium and the liver, are also vulnerable to damage caused by inflammation [73]. However, the anti-inflammatory effects of GLP-1R agonists extend well beyond adipose tissue. This was demonstrated by a study assessing the effect of liraglutide on atherothrombotic risk in obese individuals. Liraglutide was administered to 19 obese women with PCOS and 17 controls. Both groups were found to have a substantial decrease in atherothrombotic markers (such as markers of inflammation, endothelial function, and clotting) after six months of treatment [74]. Another study reported a decline in the adhesion of monocytes to cultured aortic endothelial cells treated with liraglutide; liraglutide also reduced the inflammatory response to TNFα and LPS stimulation [75].

GLP-1R agonists also exert their anti-inflammatory effects on the liver. A strong association has been reported between PCOS and no-nalcoholic fatty liver disease [76]. However, a rodent study found that liraglutide can reduce hepatic inflammation and alleviate the accumulation of M1 pro-inflammatory macrophages [77].

Multiple studies comparing different GLP-1R agonists with metformin or orlistat have been conducted to determine the most effective weight loss option for patients with PCOS. In a double-blind placebo-controlled trial, PCOS patients were administered liraglutide over 20 weeks; 76% of women lost more than 5% of their weight compared to those in the placebo group or those treated only with metformin [78]. A systematic review and meta-analysis directed towards the anti-obesity effects of GLP-1R agonists further reported evidence of higher weight reduction (weighted mean difference (WMD) = −2.61), greater waist circumference reduction (WMD = −3.46) and change in body mass index (WMD = −0.93) associated with the treatment of GLP-1R agonists [79]. Along with these studies and several additional meta-analyses, it can be concluded that GLP-1R agonists, especially liraglutide and semaglutide, are successful in inducing substantial weight reduction, are well tolerated, and also show signs of improving obesity-related risk factors [80,81].

Though GLP-1R agonists offer significant benefits in women with PCOS, they also have side effects, such as nausea, vomiting, diarrhea, constipation, and abdominal pain [82]. Other less common side effects include dyspepsia, mild tachycardia [83], nasopharyngitis, and headaches [84]. Due to their administration via injection, women with PCOS treated with long-term GLP-1R agonists, including exenatide and liraglutide, also reported experiencing injection site reactions, particularly pruritus and erythema [85,86]. However, it should be noted that these side effects are transient and should not necessarily lead to the discontinuation of the treatment. Additionally, research has shown that the advantageous weight loss effect of this incretin-based therapy reverses when the medication is stopped.

Due to the side effects observed, patients with gastroparesis, inflammatory bowel syndrome, or other gastrointestinal diseases are advised against taking GLP-1R agonists [85]. Moreover, this class of drug is also contraindicated in patients with hypersensitivity to GLP-1R agonists, pancreatic cancer, or a history of pancreatitis [87]. As reported from in vivo testing, GLP-1R agonists, specifically liraglutide, can induce hyperplasia in thyroid C cells and tumors in primates; therefore, it is recommended to avoid GLP-1R agonists in patients with a past history or family history of medullary thyroid cancer or multiple endocrine neoplasia 2 [88,89,90].

Despite the contraindications and side effects, substantial weight loss is observed due to the actions of GLP-1R agonists in PCOS patients. The proven success of this pharmaceutical class paves the way for other drugs with common mechanisms of action.

## 5. Tirzepatide

GLP-1R agonists are now common in medical practice; however, these same receptors can be further utilized by dual agonists like tirzepatide. Tirzepatide is a newly FDA-approved drug used in the management of type 2 diabetes. Along with mimicking the action of the GLP-1 hormone, this dual agonist also influences the activity of the gastric hormone glucose-dependent insulinotropic polypeptide (GIP) [91].

Tirzepatide, a 39 amino acid synthetic peptide, was synthesized by incorporating the activity of GLP-1 into the sequence of the GIP hormone [92]. This engineering of the drug resulted in a chemical structure consisting of a fatty-diacid-added analogue of GIP, which aids in enhancing the metabolism and absorption of tirzepatide. Additionally, glutamic acid units and two (2-(2-aminoethoxy)ethoxy)acetic acids are bound to the side chain of the lysine amino acid in the fatty-diacid component [93]. This structural arrangement enables tirzepatide to have a higher affinity for albumin, a plasma protein, which contributes to the drug’s longer half-life and allows it to be dosed once weekly [94]. This pharmacokinetic attribute provides a major pharmacodynamic advantage, making tirzepatide more efficacious.

### 5.1. Mechanism of Action

While previous studies have reported that GIP alone has no significant role as a therapeutic agent upon insulin levels in T2D patients, with all effects appearing to be accounted for by GLP-1, more recent research has contradicted this claim [95]. Recently, it has been shown that the incorporation of both gastrointestinal hormones, GLP-1 and GIP, has a synergistic effect. The integration of GIP and GLP-1R agonists has demonstrated a better outcome in reducing weight and blood glucose by affecting insulin concentrations compared to each hormone being administered individually [95]. Tirzepatide is classed as an imbalanced dual GLP-1/GIP agonist because it binds to both receptors with different affinities. While the molecule shows the same affinity for the GIP receptor in comparison to the GIP hormone, it binds to the GLP-1R with an affinity that is five times weaker than the GLP-1 hormone itself. This imbalanced nature may be important in the efficacy of the drug because dose escalation for GLP-1R activation may be limited by gastrointestinal side effects (like vomiting and nausea) [96], whilst GIPR activation is not associated with these side effects. A pharmacologically biased potency at the GIPR allows for full engagement within the GIP pathway while reducing GLP-1-associated tolerability problems [93] and increasing patient compliance [97].

### 5.2. GIPR Activity

GIP is a gut hormone that is produced from the K cells in the upper portion of the small intestine in response to the stimulus of a nutrient supply [98]. GIP receptors are expressed in adipose tissue, the central nervous system, and bones. GIP improves the function of white adipose tissue and has an intense anorexigenic impact through the integration of activation signals within the brain [99].

### 5.3. The Role of GIP in Glucose Metabolism

The function of GIP is to promote the secretion of nutrient-stimulated insulin and glucagon, making it a crucial factor in maintaining glucose metabolism in humans.

### 5.4. Insulin

GIP, as compared to GLP-1, has an augmented impact on the secretion of insulin. This is demonstrated by the fact that GIP accounts for 44% of the factors influencing insulin release in response to a 50 g oral glucose load, whereas glucose alone accounts for 33% and GLP-1 accounts for 22% [100]. GIP, paired with either insulin or GLP-1 signaling, causes a decline in weight and blood glucose. The involvement of GIP at the molecular level is very similar to GLP-1. It causes an increase in cAMP which ultimately increases insulin release from pancreatic beta cells while also upregulating pro-insulin gene transcription and leading to increased insulin content within beta cells [101]. However, these effects are not observed in those with insulin-resistant conditions like type 2 diabetes [102]. Additionally, the GIP hormone may play a role in facilitating the proliferation of pancreatic beta cells in response to a glucose stimulus in animals [103].

### 5.5. Glucagon

The physiological importance of GIP-induced glucagon release remains unclear. During hyperglycemic episodes, GIP does not impact the secretion of glucagon, unlike GLP-1. Neither does GIP stimulate glucagon release in those with hypoglycemia where there is normally increased glucanotropic activity as part of the counter-regulatory response [104]. The loss of GIPR activity in pancreatic beta cells has been associated with hyperglycemia and a decrease in postprandial insulin secretion. It has been suggested that GIP may also be responsible for the reduction in the expansion of pancreatic alpha cells in cases of high blood glucose levels [104].

### 5.6. The Role of GIP in Lipid Metabolism

Through the increase in blood flow in adipose tissue and uptake of triglycerides, GIP modulates fat metabolism with high efficiency, affecting energy storage and controlling fat accumulation [105]. Not only is GIP crucial in controlling food intake, but it also regulates the activity of the enzyme lipoprotein lipase, which in turn controls lipolysis within adipocytes. GIP also has lipolytic effects in states of hyperglycemia and low insulin concentration levels [105].

### 5.7. Pharmacodynamics of Tirzepatide

Tirzepatide is a subcutaneous injection that can be taken with or without food. The dosage is initiated with the lowest dose and can be increased gradually. The minimum dosage is 2.5 mg per week with a maximum of 15 mg per week and should be adjusted according to blood glucose levels. This drug aids in controlling blood glucose concentrations which, by definition, are impaired in patients with type 2 diabetes [106]. Tirzepatide is given to patients with type 2 diabetes after meals to stimulate insulin during both phases of its secretion and concurrently suppresses glucagon release, decreasing blood glucose levels [101]. Tirzepatide also increases insulin sensitivity, which aids glucose transport into cells. Additionally, the drug contributes to weight reduction by preventing gluconeogenesis by acting on the liver cells, and also slows down the transport of food in the gut, resulting in decreased food intake [107].

### 5.8. Side Effects and Contra-Indications

Tirzepatide has been shown to cause thyroid C cell tumors in animal studies. Thus, the FDA has given a boxed warning, one of the strictest warnings, to tirzepatide for thyroid C cell tumors. However, no sign of cancer has been observed in humans to date, although continuous monitoring is needed. Moreover, tirzepatide is not advised for patients with a family or personal history of thyroid cancer [91]. As the drug delays stomach emptying, it is not advisable for patients with gastroparesis. Tirzepatide can cause adverse effects like vomiting, constipation, diarrhea, and abdominal pain. Tirzepatide also interacts with drugs that have a narrow therapeutic index, such as anti-platelet drugs. Additionally, it could also affect the regulation of oral contraceptives [108].

### 5.9. Clinical Trials

Five clinical trials in diabetes have been undertaken (Surpass 1–4) [109,110,111,112,113] and the baseline characteristics are shown in Table 1.

**Figure 4:**
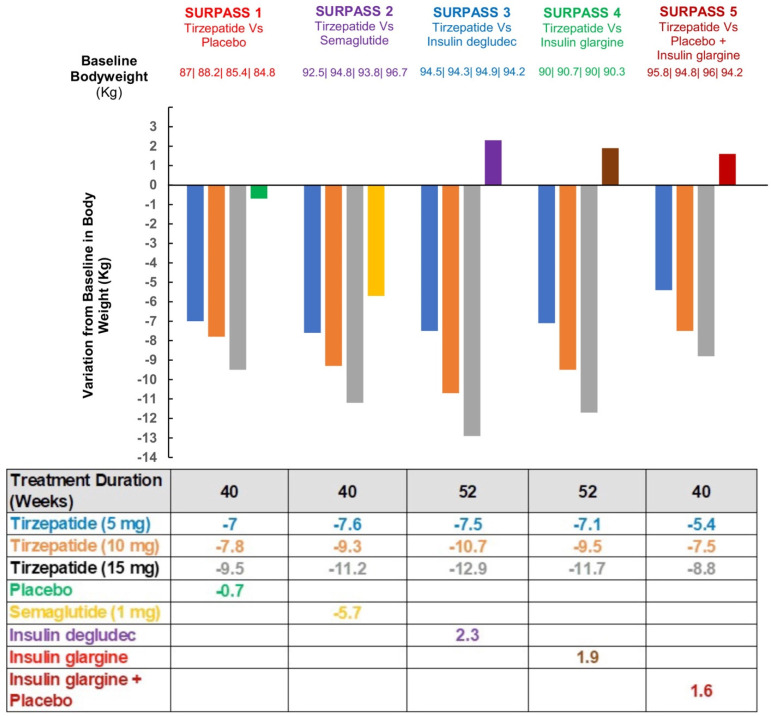
Changes from baseline body weight to end of treatment in SURPASS 1-5 [109,110,111,112,113].

In each of the studies, there was a dose-dependent reduction in HbA1c, indicating improved glycemic control in the subjects with type 2 diabetes during these 26 weeks. In addition, a marked decrease in body weight was seen with tirzepatide ranging from −0.9 to −11.3 kg, and a summary of the weight reduction for each clinical trial is shown in Figure 4. Tirzepatide has shown better results in the reduction of weight and glycated hemoglobin in patients compared to semaglutide, which appears to be the most potent out of the GLP-1RAs [111].

In addition to the weight loss reported, other beneficial results such as lipid level reductions, control in blood pressure, and fasting insulin levels improved. Additionally, obese patients without diabetes given the highest dosage of 15 mg tirzepatide over 72 weeks showed a 5% reduction in weight for approximately 95% of patients in the phase 3 trial [114]. During the treatment analysis, 40% of adults also showed at least a 25% loss in baseline weight [114]. The same clinical trial reported that patients had an increase in satiety [114]. Moreover, a trial by the American Diabetes Association supported the previous trial and noted that taking tirzepatide helped lose weight for 9 out of 10 obese adults [115,116]. Bariatric surgery results in weight loss with a 25% to 30% reduction rate over a period of 1–2 years, and recently approved drugs like tirzepatide yield approximately a 3–8% reduction on average [117].

Tirzepatide helps with weight reduction and insulin sensitivity, two of the major problems associated with PCOS. It has been stated that there is a greater probability for PCOS patients to develop T2D [118]. This is mainly due to the resistance to insulin that many PCOS patients suffer from. Tirzepatide has been proven to treat these conditions, and it can be a potential drug to be used in PCOS. Additionally, tirzepatide helps alter the gut biome favorably, which is beneficial to PCOS patients as there is an association between the gut biome and PCOS: gut dysbiosis is possibly a cause of PCOS [119]. This suggests that tirzepatide may be effective for those PCOS patients who are obese with metabolic syndrome, though not of benefit in those who are of normal weight. However, caution needs to be exercised despite the therapeutic promise that tirzepatide may have for the metabolic features of PCOS. Oral contraceptives are used in PCOS to give a regular menstrual cycle and for the treatment of hirsutism, and there is the potential that tirzepatide may interfere with their action; however, conversely, weight loss improves OCP efficacy and tirzepatide would not be expected to negatively affect alternative routes of hormonal contraceptive delivery such as vaginal or transdermal. Until confirmation that tirzepatide has no teratogenic effects, effective contraception must be used should tirzepatide be prescribed. For PCOS patients where fertility is an issue, tirzepatide is contra-indicated until more is known about its teratogenic potential. In addition, if tirzepatide is used in these young women with PCOS, it would be wise to have a washout period and not to aim for fertility, empirically, within two to three months of stopping the drug.

## 6. Additional Management Strategies in PCOS

### 6.1. Orlistat

Orlistat, a lipase inhibitor, is licensed for the management of weight in patients with obesity and has been used in the treatment of PCOS. This weight loss drug reduces lipid absorption by ~30% in the gut [120]. In a 12-week randomized open-label study, women with PCOS reported having a 4.69% decrease in weight associated with orlistat treatment [121]. Additionally, the study demonstrated that 90% of the orlistat-treated group observed a substantial weight reduction, while only 10% showed a small (0.2%) gain in weight. However, its poorly tolerated side effect profile, leading to gastrointestinal symptoms, limits its use. The majority of patients have reported experiencing steatorrhea, cramping, and diarrhea with fecal urgency and mild flatulence [122]. In addition, significant weight is only lost when lifestyle changes have been implemented concurrently alongside the intake of orlistat [123]. These side effects have decreased patient compliance, eventually leading to affected women regaining the weight initially lost due to orlistat.

### 6.2. Metformin

Metformin is the only licensed biguanide and is the primary initial medical therapy for type 2 diabetes. Metformin reduces gluconeogenesis by acting on the liver as well as adipose tissue, skeletal muscle, endothelium, and the ovaries [124]. It reduces insulin resistance and consequently is commonly used off-label for women with PCOS. However, there is significant controversy around its effectiveness in weight loss. A systematic review and meta-analysis of 543 women with PCOS found no relationship between metformin and body weight or body mass index [125]. In contrast, PCOS patients taking metformin over six months reported a mean weight loss of 2.3 kg in a randomized, controlled, double-blinded trial [126]. The latter study suggests metformin may cause moderate weight loss, but the conclusions of the randomized studies are contradictory, and metformin’s effect on body weight in PCOS appears inconsistent.

Metformin given in high doses to women with PCOS is more effective but exhibits multiple side effects. The side effects most commonly observed in PCOS patients are gastrointestinal symptoms like nausea, vomiting, diarrhea, flatulence, abdominal pain, and anorexia [127]. In addition, women with PCOS taking long-term metformin were reported to suffer from vitamin B12 deficiency, particularly in anemic or peripheral neuropathy patients [128].

Due to the lack of evidence for its efficacy and dose-dependent side effects and the fact that it may not affect all PCOS phenotypes, there are reservations for the use of metformin [127].

## 7. Bariatric Surgery

There are a number of bariatric surgery procedures, including Roux-en-Y gastric bypass, sleeve gastrectomy, adjustable gastric band, biliopancreatic diversion with duodenal switch, and single anastomosis duodeno-ileal bypass with sleeve gastrectomy; however, most randomized control trials only enroll small numbers of obese patients with PCOS and have a follow up limited to one or two years; despite these limitations, all studies report successful weight loss [129,130,131]. Patients must meet eligibility criteria for surgery, such as having a BMI greater than 35 kg/m^2^ and at least one serious complication [47]. Considering only 11.5–12.4% of PCOS patients have a BMI in the range of 35–40 kg/m^2^ [132], most are not suitable candidates for this therapy [133].

Bariatric surgery results in an increase in the release and circulation of gut peptides (including GLP-1), which may be an important driving factor for weight loss after surgery [134,135]. GLP-1 is an agonist for the GLP-1 receptor (GLP-1R), which regulates many pathways controlling fat metabolism, satiety, and pancreatic beta cell function [136]. Significant improvements in the features of PCOS post bariatric surgery were also observed in a meta-analysis involving 2130 women with a mean BMI of 48.1 kg/m^2^. A reduction (from 45.6% to 6.8%) in PCOS incidence was reported in a follow-up 12 months after surgery. Positive effects on menstrual irregularities and hirsutism were also observed [137]. However, since the surgery is expensive, invasive, and irreversible, it is not a commonly employed approach for PCOS treatment [136]. It was therefore hypothesized that using pharmacotherapy that mechanistically mimics surgery may be a more cost-effective alternative [136].

## 8. Conclusions

PCOS affects androgen production and metabolism, leading to insulin resistance, metabolic disorders, and impaired mental health. These abnormalities could further affect weight and reproductive function in adults [138]. Weight gain is a major problem in women with PCOS that exacerbates health conditions such as diabetes and cardiovascular disorders and worsens anovulation and disrupted gonadotropin production. Weight loss has proven to be beneficial in decreasing these abnormalities. Several treatments, such as lifestyle alteration, various pharmaceutical agents, and bariatric surgery, have all been considered in managing PCOS. Although GLP-1R agonists have been shown to be the most successful agents for weight loss, these drugs also have their limitations. Tirzepatide is a newly approved FDA drug that significantly reduces weight loss and improves insulin sensitivity in patients with and without diabetes. While this drug has several gastrointestinal side effects, there is a reduction in their intensity (relative to GLP-1RA drugs alone) due to its dual mechanism of action. Tirzepatide could be a novel therapeutic strategy for PCOS, particularly in addressing weight gain and insulin sensitivity. However, to confirm this, robust clinical trials will need to be undertaken with tirzepatide in PCOS patients. Further, such clinical trials should incorporate subjects of differing ethnicities.

## Figures and Tables

**Table 1:** Baseline clinical trial design for SURPASS 1-5 [109,110,111,112,113].

Trial	SURPASS 1			SURPASS 2			SURPASS 3			SURPASS 4			SURPASS 5		
**Tirzepatide**	5 mg/10 mg/15 mg			5 mg/10 mg/15 mg			5 mg/10 mg/15 mg			5 mg/10 mg/15 mg			5 mg/10 mg/15 mg		
**Comparator**	Placebo			Semaglutide 1.0 mg			Insulin degludec			Insulin glargine			Placebo + Insulin glargine		
**Trial duration**	40 weeks			40 weeks			52 weeks			52 weeks			40 weeks		
**Design**	double-blind placebo-controlled			open-label			open-label			open-label			double-blind placebo-controlled		
**Tirzepatide**	5 mg	10 mg	15 mg	5 mg	10 mg	15 mg	5 mg	10 mg	15 mg	5 mg	10 mg	15 mg	5 mg	10 mg	15 mg
**Patients number**	121	121	121	470	469	470	358	360	359	329	328	338	116	119	120

## Data Availability

There is no original data within this review.

## References

[1] Himelein M.J., Thatcher S.S. (2006). Polycystic ovary syndrome and mental health: A review. Obstet. Gynecol. Surv..

[2] Shroff R., Kerchner A., Maifeld M., Van Beek E.J., Jagasia D., Dokras A. (2007). Young obese women with polycystic ovary syndrome have evidence of early coronary atherosclerosis. J. Clin. Endocrinol. Metab..

[3] Legro R.S., Arslanian S.A., Ehrmann D.A., Hoeger K.M., Murad M.H., Pasquali R., Welt C.K. (2013). Diagnosis and treatment of polycystic ovary syndrome: An Endocrine Society clinical practice guideline. J. Clin. Endocrinol. Metab..

[4] Deswal R., Narwal V., Dang A., Pundir C.S. (2020). The Prevalence of Polycystic Ovary Syndrome: A Brief Systematic Review. J. Hum. Reprod. Sci..

[5] Diamanti-Kandarakis E., Kouli C.R., Bergiele A.T., Filandra F.A., Tsianateli T.C., Spina G.G., Zapanti E.D., Bartzis M.I. (1999). A survey of the polycystic ovary syndrome in the Greek island of Lesbos: Hormonal and metabolic profile. J. Clin. Endocrinol. Metab..

[6] Chaney P. Are the Rotterdam Criteria Still Relevant in PCOS Diagnoses?. https://www.volusonclub.net/empowered-womens-health/are-the-rotterdam-criteria-still-relevant-in-pcos-diagnoses-weighing-the-consensus-current-relevance/.

[7] Teede H., Deeks A., Moran L. (2010). Polycystic ovary syndrome: A complex condition with psychological, reproductive and metabolic manifestations that impacts on health across the lifespan. BMC Med..

[8] Smet M.E., McLennan A. (2018). Rotterdam criteria, the end. Australas. J. Ultrasound Med..

[9] Torpy J.M., Lynm C., Glass R.M. (2007). Polycystic Ovary Syndrome. JAMA.

[10] Rosenfield R.L., Ehrmann D.A. (2016). The Pathogenesis of Polycystic Ovary Syndrome (PCOS): The Hypothesis of PCOS as Functional Ovarian Hyperandrogenism Revisited. Endocr. Rev..

[11] Cleveland Clinic Androgens: Function, Measurement and Related Disorders. https://my.clevelandclinic.org/health/articles/22002-androgens.

[12] Goodarzi M.O., Carmina E., Azziz R. (2015). DHEA, DHEAS and PCOS. J. Steroid Biochem. Mol. Biol..

[13] Legro R.S. (2015). Evaluation and Treatment of Polycystic Ovary Syndrome.

[14] Ashraf S., Nabi M., Rasool S.U.A., Rashid F., Amin S. (2019). Hyperandrogenism in polycystic ovarian syndrome and role of CYP gene variants: A review. Egypt. J. Med. Hum. Genet..

[15] Gainder S., Sharma B. (2019). Update on Management of Polycystic Ovarian Syndrome for Dermatologists. Indian Derm. Online J..

[16] Cooney L.G., Lee I., Sammel M.D., Dokras A. (2017). High prevalence of moderate and severe depressive and anxiety symptoms in polycystic ovary syndrome: A systematic review and meta-analysis. Hum. Reprod..

[17] Diamanti-Kandarakis E., Dunaif A. (2012). Insulin resistance and the polycystic ovary syndrome revisited: An update on mechanisms and implications. Endocr. Rev..

[18] Galan N. Symptoms and Screening of Insulin Resistance with PCOS. https://www.verywellhealth.com/pcos-and-insulin-resistance-2616319.

[19] Goodarzi M.O., Korenman S.G. (2003). The importance of insulin resistance in polycystic ovary syndrome. Fertil. Steril..

[20] Wilcox G. (2005). Insulin and insulin resistance. Clin. Biochem. Rev..

[21] Sinha S.K., Chan K.K.-C., Sadeghi-Nejad A. (2022). Hyperinsulinism: Background, Pathophysiology, Etiology.

[22] Baptiste C.G., Battista M.C., Trottier A., Baillargeon J.P. (2010). Insulin and hyperandrogenism in women with polycystic ovary syndrome. J. Steroid Biochem. Mol. Biol..

[23] Cena H., Chiovato L., Nappi R.E. (2020). Obesity, Polycystic Ovary Syndrome, and Infertility: A New Avenue for GLP-1 Receptor Agonists. J. Clin. Endocrinol. Metab..

[24] Legro R.S., Castracane V.D., Kauffman R.P. (2004). Detecting insulin resistance in polycystic ovary syndrome: Purposes and pitfalls. Obs. Gynecol. Surv..

[25] Barber T.M., Hanson P., Weickert M.O., Franks S. (2019). Obesity and Polycystic Ovary Syndrome: Implications for Pathogenesis and Novel Management Strategies. Clin. Med. Insights Reprod. Health.

[26] Teede H.J., Misso M.L., Costello M.F., Dokras A., Laven J., Moran L., Piltonen T., Norman R.J. (2018). Recommendations from the international evidence-based guideline for the assessment and management of polycystic ovary syndrome. Hum. Reprod..

[27] Joshi M., Shankar R., Pathak K., Yadav R. (2021). Polycystic ovarian syndrome: A review covering phytoconstituents for its outstrip management. Pharmacol. Res. Mod. Chin. Med..

[28] Fraison E., Kostova E., Moran L.J., Bilal S., Ee C.C., Venetis C., Costello M.F. (2020). Metformin versus the combined oral contraceptive pill for hirsutism, acne, and menstrual pattern in polycystic ovary syndrome. Cochrane Database Syst. Rev..

[29] Chavda V.P., Ajabiya J., Teli D., Bojarska J., Apostolopoulos V. (2022). Tirzepatide, a New Era of Dual-Targeted Treatment for Diabetes and Obesity: A Mini-Review. Molecules.

[30] Sloop K.W., Briere D.A., Emmerson P.J., Willard F.S. (2018). Beyond Glucagon-like Peptide-1: Is G-Protein Coupled Receptor Polypharmacology the Path Forward to Treating Metabolic Diseases?. ACS Pharm. Transl. Sci..

[31] Norman R.J., Teede H.J. (2018). A new evidence-based guideline for assessment and management of polycystic ovary syndrome. Med. J. Aust..

[32] Kim C.H., Lee S.H. (2022). Effectiveness of Lifestyle Modification in Polycystic Ovary Syndrome Patients with Obesity: A Systematic Review and Meta-Analysis. Life.

[33] Papavasiliou K., Papakonstantinou E. (2017). Nutritional support and dietary interventions for women with polycystic ovary syndrome. Nutr. Diet. Suppl..

[34] Wong J.M., Gallagher M., Gooding H., Feldman H.A., Gordon C.M., Ludwig D.S., Ebbeling C.B. (2016). A randomized pilot study of dietary treatments for polycystic ovary syndrome in adolescents. Pediatr. Obes..

[35] Moran L.J., Hutchison S.K., Norman R.J., Teede H.J. (2011). Lifestyle changes in women with polycystic ovary syndrome. Cochrane Database Syst. Rev..

[36] Patel S. (2018). Polycystic ovary syndrome (PCOS), an inflammatory, systemic, lifestyle endocrinopathy. J. Steroid Biochem. Mol. Biol..

[37] Kose O., Arabaci T., Kara A., Yemenoglu H., Kermen E., Kizildag A., Gedikli S., Ozkanlar S. (2016). Effects of Melatonin on Oxidative Stress Index and Alveolar Bone Loss in Diabetic Rats With Periodontitis. J. Periodontol..

[38] Lim S., Smith C.A., Costello M.F., MacMillan F., Moran L., Ee C. (2019). Barriers and facilitators to weight management in overweight and obese women living in Australia with PCOS: A qualitative study. BMC Endocr. Disord..

[39] Domecq J.P., Prutsky G., Mullan R.J., Hazem A., Sundaresh V., Elamin M.B., Phung O.J., Wang A., Hoeger K., Pasquali R. (2013). Lifestyle modification programs in polycystic ovary syndrome: Systematic review and meta-analysis. J. Clin. Endocrinol. Metab..

[40] Hill J.O., Wyatt H.R., Peters J.C. (2013). The Importance of Energy Balance. Eur. Endocrinol..

[41] Dalla Man C., Micheletto F., Sathananthan A., Rizza R.A., Vella A., Cobelli C. (2010). A model of GLP-1 action on insulin secretion in nondiabetic subjects. Am. J. Physiol. Endocrinol. Metab..

[42] Mehta A., Marso S.P., Neeland I.J. (2017). Liraglutide for weight management: A critical review of the evidence. Obes. Sci. Prac..

[43] Dailey M.J., Moran T.H. (2013). Glucagon-like peptide 1 and appetite. Trends Endocrinol. Metab..

[44] Sisley S., Gutierrez-Aguilar R., Scott M., D’Alessio D.A., Sandoval D.A., Seeley R.J. (2014). Neuronal GLP1R mediates liraglutide’s anorectic but not glucose-lowering effect. J. Clin. Investig..

[45] Knudsen L.B., Lau J. (2019). The Discovery and Development of Liraglutide and Semaglutide. Front. Endocrinol..

[46] van Bloemendaal L., Ten Kulve J.S., la Fleur S.E., Ijzerman R.G., Diamant M. (2014). Effects of glucagon-like peptide 1 on appetite and body weight: Focus on the CNS. J. Endocrinol..

[47] Bednarz K., Kowalczyk K., Cwynar M., Czapla D., Czarkowski W., Kmita D., Nowak A., Madej P. (2022). The Role of Glp-1 Receptor Agonists in Insulin Resistance with Concomitant Obesity Treatment in Polycystic Ovary Syndrome. Int. J. Mol. Sci..

[48] Zhao L., Zhu C., Lu M., Chen C., Nie X., Abudukerimu B., Zhang K., Ning Z., Chen Y., Cheng J. (2019). The key role of a glucagon-like peptide-1 receptor agonist in body fat redistribution. J. Endocrinol..

[49] Ishii S., Nagai Y., Sada Y., Fukuda H., Nakamura Y., Matsuba R., Nakagawa T., Kato H., Tanaka Y. (2019). Liraglutide Reduces Visceral and Intrahepatic Fat Without Significant Loss of Muscle Mass in Obese Patients With Type 2 Diabetes: A Prospective Case Series. J. Clin. Med. Res..

[50] Yaribeygi H., Sathyapalan T., Sahebkar A. (2019). Molecular mechanisms by which GLP-1 RA and DPP-4i induce insulin sensitivity. Life Sci..

[51] Challa T.D., Beaton N., Arnold M., Rudofsky G., Langhans W., Wolfrum C. (2012). Regulation of adipocyte formation by GLP-1/GLP-1R signaling. J. Biol. Chem..

[52] Chen J., Zhao H., Ma X., Zhang Y., Lu S., Wang Y., Zong C., Qin D., Wang Y., Yingfeng Yang Y. (2017). GLP-1/GLP-1R Signaling in Regulation of Adipocyte Differentiation and Lipogenesis. Cell. Physiol. Biochem..

[53] Li H., Donelan W., Wang F., Zhang P., Yang L., Ding Y., Tang D., Li S. (2021). GLP-1 Induces the Expression of FNDC5 Derivatives That Execute Lipolytic Actions. Front. Cell Dev. Biol..

[54] Sulaiman M.A., Al-Farsi Y.M., Al-Khaduri M.M., Saleh J., Waly M.I. (2018). Polycystic ovarian syndrome is linked to increased oxidative stress in Omani women. Int. J. Womens Health.

[55] Betteridge D.J. (2000). What is oxidative stress?. Metabolism.

[56] Yaribeygi H., Sathyapalan T., Atkin S.L., Sahebkar A. (2020). Molecular Mechanisms Linking Oxidative Stress and Diabetes Mellitus. Oxid. Med. Cell. Longev..

[57] Michos E.D. Polycystic Ovarian Syndrome: How Your Ovaries Can Affect Your Heart. https://www.hopkinsmedicine.org/health/conditions-and-diseases/polycystic-ovarian-syndrome-how-your-ovaries-can-affect-your-heart.

[58] Antipolis S. Young Women with Polycystic Ovary Syndrome Have Raised Risk of Heart Disease. https://www.escardio.org/The-ESC/Press-Office/Press-releases/Young-women-with-polycystic-ovary-syndrome-have-raised-risk-of-heart-disease.

[59] Rizzo M., Abate N., Chandalia M., Rizvi A.A., Giglio R.V., Nikolic D., Marino Gammazza A., Barbagallo I., Isenovic E.R., Banach M. (2015). Liraglutide reduces oxidative stress and restores heme oxygenase-1 and ghrelin levels in patients with type 2 diabetes: A prospective pilot study. J. Clin. Endocrinol. Metab..

[60] Fernández-Millán E., Martín M.A., Goya L., Lizárraga-Mollinedo E., Escrivá F., Ramos S., Álvarez C. (2016). Glucagon-like peptide-1 improves beta-cell antioxidant capacity via extracellular regulated kinases pathway and Nrf2 translocation. Free. Radic. Biol. Med..

[61] He F., Ru X., Wen T. (2020). NRF2, a Transcription Factor for Stress Response and Beyond. Int. J. Mol. Sci..

[62] Cai X., She M., Xu M., Chen H., Li J., Chen X., Zheng D., Liu J., Chen S., Zhu J. (2018). GLP-1 treatment protects endothelial cells from oxidative stress-induced autophagy and endothelial dysfunction. Int. J. Biol. Sci..

[63] Frostegård J. (2013). Immunity, atherosclerosis and cardiovascular disease. BMC Med..

[64] Sathyapalan T., Atkin S.L. (2010). Mediators of inflammation in polycystic ovary syndrome in relation to adiposity. Mediat. Inflamm..

[65] Lee Y.S., Jun H.S. (2016). Anti-Inflammatory Effects of GLP-1-Based Therapies beyond Glucose Control. Mediat. Inflamm..

[66] Kanda H., Tateya S., Tamori Y., Kotani K., Hiasa K., Kitazawa R., Kitazawa S., Miyachi H., Maeda S., Egashira K. (2006). MCP-1 contributes to macrophage infiltration into adipose tissue, insulin resistance, and hepatic steatosis in obesity. J. Clin. Investig..

[67] Lee Y.S., Park M.S., Choung J.S., Kim S.S., Oh H.H., Choi C.S., Ha S.Y., Kang Y., Kim Y., Jun H.S. (2012). Glucagon-like peptide-1 inhibits adipose tissue macrophage infiltration and inflammation in an obese mouse model of diabetes. Diabetologia.

[68] Giuliani C., Bucci I., Napolitano G. (2018). The Role of the Transcription Factor Nuclear Factor-kappa B in Thyroid Autoimmunity and Cancer. Front. Endocrinol..

[69] Guo C., Huang T., Chen A., Chen X., Wang L., Shen F., Gu X. (2016). Glucagon-like peptide 1 improves insulin resistance in vitro through anti-inflammation of macrophages. Braz. J. Med. Biol. Res..

[70] Kim Chung le T., Hosaka T., Yoshida M., Harada N., Sakaue H., Sakai T., Nakaya Y. (2009). Exendin-4, a GLP-1 receptor agonist, directly induces adiponectin expression through protein kinase A pathway and prevents inflammatory adipokine expression. Biochem. Biophys. Res. Commun..

[71] Ouchi N., Walsh K. (2007). Adiponectin as an anti-inflammatory factor. Clin. Chim. Acta.

[72] Dawson A.J., Sathyapalan T., Vince R., Coady A.M., Ajjan R.A., Kilpatrick E.S., Atkin S.L. (2019). The Effect of Exenatide on Cardiovascular Risk Markers in Women With Polycystic Ovary Syndrome. Front. Endocrinol..

[73] Foltyn W., Strzelczyk J., Marek B., Kajdaniuk D., Siemińska L., Zemczak A., Blicharz-Dorniak J., Kos-Kudła B. (2011). Selected markers of endothelial dysfunction in women with polycystic ovary syndrome. Endokrynol. Pol..

[74] Kahal H., Aburima A., Ungvari T., Rigby A.S., Coady A.M., Vince R.V., Ajjan R.A., Kilpatrick E.S., Naseem K.M., Atkin S.L. (2015). The effects of treatment with liraglutide on atherothrombotic risk in obese young women with polycystic ovary syndrome and controls. BMC Endocr. Disord..

[75] Krasner N.M., Ido Y., Ruderman N.B., Cacicedo J.M. (2014). Glucagon-like peptide-1 (GLP-1) analog liraglutide inhibits endothelial cell inflammation through a calcium and AMPK dependent mechanism. PLoS ONE.

[76] Vassilatou E. (2014). Nonalcoholic fatty liver disease and polycystic ovary syndrome. World J. Gastroenterol..

[77] Somm E., Montandon S.A., Loizides-Mangold U., Gaïa N., Lazarevic V., De Vito C., Perroud E., Bochaton-Piallat M.-L., Dibner C., Schrenzel J. (2021). The GLP-1R agonist liraglutide limits hepatic lipotoxicity and inflammatory response in mice fed a methionine-choline deficient diet. Transl. Res..

[78] Astrup A., Rössner S., Van Gaal L., Rissanen A., Niskanen L., Al Hakim M., Madsen J., Rasmussen M.F., Lean M.E. (2009). Effects of liraglutide in the treatment of obesity: A randomised, double-blind, placebo-controlled study. Lancet.

[79] Lyu X., Lyu T., Wang X., Zhu H., Pan H., Wang L., Yang H., Gong F. (2021). The Antiobesity Effect of GLP-1 Receptor Agonists Alone or in Combination with Metformin in Overweight/Obese Women with Polycystic Ovary Syndrome: A Systematic Review and Meta-Analysis. Int. J. Endocrinol..

[80] Ma R., Ding X., Wang Y., Deng Y., Sun A. (2021). The therapeutic effects of glucagon-like peptide-1 receptor agonists and metformin on polycystic ovary syndrome: A protocol for systematic review and meta-analysis. Medicine.

[81] Abdalla M.A., Deshmukh H., Atkin S., Sathyapalan T. (2021). The potential role of incretin-based therapies for polycystic ovary syndrome: A narrative review of the current evidence. Ther. Adv. Endocrinol. Metab..

[82] Sun F., Chai S., Yu K., Quan X., Yang Z., Wu S., Zhang Y., Ji L., Wang J., Shi L. (2015). Gastrointestinal adverse events of glucagon-like peptide-1 receptor agonists in patients with type 2 diabetes: A systematic review and network meta-analysis. Diabetes Technol..

[83] Ratner R., Han J., Nicewarner D., Yushmanova I., Hoogwerf B.J., Shen L. (2011). Cardiovascular safety of exenatide BID: An integrated analysis from controlled clinical trials in participants with type 2 diabetes. Cardiovasc. Diabetol..

[84] Filippatos T.D., Panagiotopoulou T.V., Elisaf M.S. (2014). Adverse Effects of GLP-1 Receptor Agonists. Rev. Diabet. Stud..

[85] Collins L., Costello R.A. (2022). Glucagon-Like Peptide-1 Receptor Agonists.

[86] Garber A.J. (2011). Long-acting glucagon-like peptide 1 receptor agonists: A review of their efficacy and tolerability. Diabetes Care.

[87] Anderson S.L., Trujillo J.M. (2010). Association of pancreatitis with glucagon-like peptide-1 agonist use. Ann. Pharm..

[88] Bjerre Knudsen L., Madsen L.W., Andersen S., Almholt K., de Boer A.S., Drucker D.J., Gotfredsen C., Egerod F.L., Hegelund A.C., Jacobsen H. (2010). Glucagon-like Peptide-1 receptor agonists activate rodent thyroid C-cells causing calcitonin release and C-cell proliferation. Endocrinology.

[89] Boess F., Bertinetti-Lapatki C., Zoffmann S., George C., Pfister T., Roth A., Lee S.M., Thasler W.E., Singer T., Suter L. (2013). Effect of GLP1R agonists taspoglutide and liraglutide on primary thyroid C-cells from rodent and man. J. Mol. Endocrinol..

[90] Chiu W.Y., Shih S.-R., Tseng C.-H. (2012). A review on the association between glucagon-like peptide-1 receptor agonists and thyroid cancer. Exp. Diabetes Res..

[91] FDA FDA Approves Novel, Dual-Targeted Treatment for Type 2 Diabetes. https://www.fda.gov/news-events/press-announcements/fda-approves-novel-dual-targeted-treatment-type-2-diabetes.

[92] Coskun T., Sloop K.W., Loghin C., Alsina-Fernandez J., Urva S., Bokvist K.B., Cui X., Briere D.A., Cabrera O., Roell W.C. (2018). LY3298176, a novel dual GIP and GLP-1 receptor agonist for the treatment of type 2 diabetes mellitus: From discovery to clinical proof of concept. Mol. Metab..

[93] Willard F.S., Douros J.D., Gabe M.B., Showalter A.D., Wainscott D.B., Suter T.M., Capozzi M.E., van der Velden W.J., Stutsman C., Cardona G.R. (2020). Tirzepatide is an imbalanced and biased dual GIP and GLP-1 receptor agonist. JCI Insight.

[94] Østergaard S., Paulsson J.F., Kofoed J., Zosel F., Olsen J., Jeppesen C.B., Spetzler J., Ynddal L., Schleiss L.G., Christoffersen B.Ø. (2021). The effect of fatty diacid acylation of human PYY_3-36_ on Y_2_ receptor potency and half-life in minipigs. Sci. Rep..

[95] Min T., Bain S.C. (2021). The Role of Tirzepatide, Dual GIP and GLP-1 Receptor Agonist, in the Management of Type 2 Diabetes: The SURPASS Clinical Trials. Diabetes.

[96] Andersen A., Lund A., Knop F.K., Vilsbøll T. (2018). Glucagon-like peptide 1 in health and disease. Nat. Rev. Endocrinol..

[97] Roose S.P. (2003). Compliance: The impact of adverse events and tolerability on the physician’s treatment decisions. Eur. Neuropsychopharmacol..

[98] Usdin T.B., Mezey E., Button D.C., Brownstein M.J., Bonner T.I. (1993). Gastric inhibitory polypeptide receptor, a member of the secretin-vasoactive intestinal peptide receptor family, is widely distributed in peripheral organs and the brain. Endocrinology.

[99] Kaneko S. (2022). Tirzepatide: A Novel, Once-weekly Dual GIP and GLP-1 Receptor Agonist for the Treatment of Type 2 Diabetes. Touchrev. Endocrinol..

[100] Nauck M.A., Meier J.J. (2019). GIP and GLP-1: Stepsiblings Rather Than Monozygotic Twins Within the Incretin Family. Diabetes.

[101] Ali R., Virendra S.A., Chawla P.A. (2022). Bumps and humps in the success of Tirzepatide as the first GLP1 and GIP receptor agonist. Health Sci. Rev..

[102] Boer G.A.-O.X., Holst J.A.-O. (2020). Incretin Hormones and Type 2 Diabetes-Mechanistic Insights and Therapeutic Approaches. Biology.

[103] Kim S.J., Winter K., Nian C., Tsuneoka M., Koda Y., McIntosh C.H. (2005). Glucose-dependent insulinotropic polypeptide (GIP) stimulation of pancreatic beta-cell survival is dependent upon phosphatidylinositol 3-kinase (PI3K)/protein kinase B (PKB) signaling, inactivation of the forkhead transcription factor Foxo1, and down-regulation of bax expression. J. Biol. Chem..

[104] Christensen M.B., Calanna S., Holst J.J., Vilsbøll T., Knop F.K. (2014). Glucose-dependent insulinotropic polypeptide: Blood glucose stabilizing effects in patients with type 2 diabetes. J. Clin. Endocrinol. Metab..

[105] Harada N., Hamasaki A., Yamane S., Muraoka A., Joo E., Fujita K., Inagaki N. (2011). Plasma gastric inhibitory polypeptide and glucagon-like peptide-1 levels after glucose loading are associated with different factors in Japanese subjects. J. Diabetes Investig..

[106] Seino Y., Fukushima M., Yabe D. (2010). GIP and GLP-1, the two incretin hormones: Similarities and differences. J. Diabetes Investig..

[107] Gastaldelli A., Stefan N., Häring H.U. (2021). Liver-targeting drugs and their effect on blood glucose and hepatic lipids. Diabetologia.

[108] FDA (2022). Highlights of Prescribing Information.

[109] Dahl D., Onishi Y., Norwood P., Huh R., Bray R., Patel H., Rodríguez Á. (2022). Effect of Subcutaneous Tirzepatide vs. Placebo Added to Titrated Insulin Glargine on Glycemic Control in Patients With Type 2 Diabetes: The SURPASS-5 Randomized Clinical Trial. JAMA.

[110] Del Prato S., Kahn S.E., Pavo I., Weerakkody G.J., Yang Z., Doupis J., Aizenberg D., Wynne A.G., Riesmeyer J.S., Heine R.J. (2021). Tirzepatide versus insulin glargine in type 2 diabetes and increased cardiovascular risk (SURPASS-4): A randomised, open-label, parallel-group, multicentre, phase 3 trial. Lancet.

[111] Frías J.P., Davies M.J., Rosenstock J., Pérez Manghi F.C., Fernández Landó L., Bergman B.K., Liu B., Cui X., Brown K. (2021). Tirzepatide versus Semaglutide Once Weekly in Patients with Type 2 Diabetes. N. Engl. J. Med..

[112] Ludvik B., Giorgino F., Jódar E., Frias J.P., Fernández Landó L., Brown K., Bray R., Rodríguez Á. (2021). Once-weekly tirzepatide versus once-daily insulin degludec as add-on to metformin with or without SGLT2 inhibitors in patients with type 2 diabetes (SURPASS-3): A randomised, open-label, parallel-group, phase 3 trial. Lancet.

[113] Rosenstock J., Wysham C., Frías J.P., Kaneko S., Lee C.J., Fernández Landó L., Mao H., Cui X., Karanikas C.A., Thieu V.T. (2021). Efficacy and safety of a novel dual GIP and GLP-1 receptor agonist tirzepatide in patients with type 2 diabetes (SURPASS-1): A double-blind, randomised, phase 3 trial. Lancet.

[114] Zoler M.L. Tirzepatide Powers ‘Unprecedented’ Weight Loss in Obesity Trial. https://www.medscape.com/viewarticle/975061.

[115] ADA SURMOUNT-1 Study Finds Individuals with Obesity Lost Up to 22.5% of Their Body Weight when Taking Tirzepatide. https://diabetes.org/newsroom/press-releases/2022/surmount-1-study-finds-individuals-%20with-obesity-lost-up-to-22.5-percent-body-weight-taking-tirzepatide.

[116] Jastreboff A.M., Aronne L.J., Ahmad N.N., Wharton S., Connery L., Alves B., Kiyosue A., Zhang S., Liu B., Bunck M.C. (2022). Tirzepatide Once Weekly for the Treatment of Obesity. N. Engl. J. Med..

[117] Mazin A. Drug Leads to Drastic Weight Loss with Diet and Exercise. https://www.lifespan.io/news/drug-leads-to-drastic-weight-loss-with-diet-and-exercise/.

[118] Kakoly N.S., Khomami M.B., Joham A.E., Cooray S.D., Misso M.L., Norman R.J., Harrison C.L., Ranasinha S., Teede H.J., Moran L.J. (2018). Ethnicity, obesity and the prevalence of impaired glucose tolerance and type 2 diabetes in PCOS: A systematic review and meta-regression. Hum. Reprod. Update.

[119] Sacerdote A., Zhengchao W. (2022). Rare and Underappreciated Causes of Polycystic Ovarian Syndrome. Polycystic Ovary Syndrome.

[120] Guerciolini R. (1997). Mode of action of orlistat. Int. J. Obes. Relat. Metab. Disord..

[121] Jayagopal V., Kilpatrick E.S., Holding S., Jennings P.E., Atkin S.L. (2005). Orlistat is as beneficial as metformin in the treatment of polycystic ovarian syndrome. J. Clin. Endocrinol. Metab..

[122] Graff S.K., Mario F.M., Ziegelmann P., Spritzer P.M. (2016). Effects of orlistat vs. metformin on weight loss-related clinical variables in women with PCOS: Systematic review and meta-analysis. Int. J. Clin. Prac..

[123] Panidis D., Tziomalos K., Papadakis E., Chatzis P., Kandaraki E.A., Tsourdi E.A., Katsikis I. (2014). The role of orlistat combined with lifestyle changes in the management of overweight and obese patients with polycystic ovary syndrome. Clin. Endocrinol..

[124] Setter S.M., Iltz J.L., Thams J., Campbell R.K. (2003). Metformin hydrochloride in the treatment of type 2 diabetes mellitus: A clinical review with a focus on dual therapy. Clin. Ther..

[125] Lord J.M., Flight I.H., Norman R.J. (2003). Metformin in polycystic ovary syndrome: Systematic review and meta-analysis. BMJ.

[126] Trolle B., Flyvbjerg A., Kesmodel U., Lauszus F.F. (2007). Efficacy of metformin in obese and non-obese women with polycystic ovary syndrome: A randomized, double-blinded, placebo-controlled cross-over trial. Hum. Reprod..

[127] Lashen H. (2010). Review: Role of metformin in the management of polycystic ovary syndrome. Ther. Adv. Endocrinol. Metab..

[128] Aroda V.R., Edelstein S.L., Goldberg R.B., Knowler W.C., Marcovina S.M., Orchard T.J., Bray G.A., Schade D.S., Temprosa M.G., White N.H. (2016). Long-term Metformin Use and Vitamin B12 Deficiency in the Diabetes Prevention Program Outcomes Study. J. Clin. Endocrinol. Metab..

[129] Rives-Lange C., Rassy N., Carette C., Phan A., Barsamian C., Thereaux J., Moszkowicz D., Poghosyan T., Czernichow S. (2022). Seventy years of bariatric surgery: A systematic mapping review of randomized controlled trials. Obes. Rev. Off. J. Int. Assoc. Study Obes..

[130] Lee R., Joy Mathew C., Jose M.T., Elshaikh A.O., Shah L., Cancarevic I. (2020). A Review of the Impact of Bariatric Surgery in Women With Polycystic Ovary Syndrome. Cureus.

[131] SIGN Management of Obesity: A National Clinical Guideline. https://www.sign.ac.uk/assets/sign115.pdf.

[132] Yildiz B.O., Knochenhauer E.S., Azziz R. (2008). Impact of obesity on the risk for polycystic ovary syndrome. J. Clin. Endocrinol. Metab..

[133] Johansson K., Cnattingius S., Näslund I., Roos N., Trolle Lagerros Y., Granath F., Stephansson O., Neovius M. (2015). Outcomes of pregnancy after bariatric surgery. N. Engl. J. Med..

[134] Meek C.L., Lewis H.B., Reimann F., Gribble F.M., Park A.J. (2016). The effect of bariatric surgery on gastrointestinal and pancreatic peptide hormones. Peptides.

[135] Moffett R.C., Naughton V. (2020). Emerging role of GIP and related gut hormones in fertility and PCOS. Peptides.

[136] Khan D., Moffett R.C. (2020). Commentary: Emerging role of GIP and related gut hormones in fertility and PCOS. J. Endocrinol. Sci..

[137] Skubleny D., Switzer N.J., Gill R.S., Dykstra M., Shi X., Sagle M.A., de Gara C., Birch D.W., Karmali S. (2016). The Impact of Bariatric Surgery on Polycystic Ovary Syndrome: A Systematic Review and Meta-analysis. Obes. Surg..

[138] Pasquali R., Stener-Victorin E., Yildiz B.O., Duleba A.J., Hoeger K., Mason H., Homburg R., Hickey T., Franks S., Tapanainen J.S. (2011). PCOS Forum: Research in polycystic ovary syndrome today and tomorrow. Clin. Endocrinol..

